# Transcriptional mechanisms for differential expression of outer membrane cytochrome genes *omcA* and *mtrC* in *Shewanella oneidensis* MR-1

**DOI:** 10.1186/s12866-015-0406-8

**Published:** 2015-03-21

**Authors:** Takuya Kasai, Atsushi Kouzuma, Hideaki Nojiri, Kazuya Watanabe

**Affiliations:** School of Life Sciences, Tokyo University of Pharmacy and Life Sciences, 1432-1 Horinouchi, Hachioji, 192-0392 Tokyo Japan; Biotechnology Research Center, The University of Tokyo, 1-1-1 Yayoi, Bunkyo-ku, 113-8657 Tokyo Japan

**Keywords:** Extracellular electron transfer, Outer membrane cytochrome, Transcriptional regulation, *Shewanella*

## Abstract

**Background:**

*Shewanella oneidensis* MR-1 is capable of reducing extracellular electron acceptors, such as metals and electrodes, through the Mtr respiratory pathway, which consists of the outer membrane cytochromes OmcA and MtrC and associated proteins MtrA and MtrB. These proteins are encoded in the *mtr* gene cluster (*omcA*-*mtrCAB*) in the MR-1 chromosome.

**Results:**

Here, we investigated the transcriptional mechanisms for the *mtr* genes and demonstrated that *omcA* and *mtrC* are transcribed from two upstream promoters, P_*omcA*_ and P_*mtrC*_, respectively. *In vivo* transcription and *in vitro* electrophoretic mobility shift assays revealed that a cAMP receptor protein (CRP) positively regulates the expression of the *mtr* genes by binding to the upstream regions of P_*omcA*_ and P_*mtrC*_. However, the expression of *omcA* and *mtrC* was differentially regulated in response to culture conditions; specifically, the expression from P_*mtrC*_ was higher under aerobic conditions than that under anaerobic conditions with fumarate as an electron acceptor, whereas expression from P_*omcA*_ exhibited the opposite trend. Deletion of the region upstream of the CRP-binding site of P_*omcA*_ resulted in a significant increase in promoter activity under aerobic conditions, demonstrating that the deleted region is involved in the negative regulation of P_*omcA*_.

**Conclusions:**

Taken together, the present results indicate that transcription of the *mtr* genes is regulated by multiple promoters and regulatory systems, including the CRP/cAMP-dependent regulatory system and yet-unidentified negative regulators.

**Electronic supplementary material:**

The online version of this article (doi:10.1186/s12866-015-0406-8) contains supplementary material, which is available to authorized users.

## Background

*Shewanella* species belong to the class *Gammaproteobacteria* and are widely distributed in nature, including marine and freshwater sediments [1,2]. A few members of this genus have attracted considerable attention due to their importance in the biogeochemical cycling of metals [3] and utility in biotechnology processes, such as bioremediation [4] and bioelectrochemical systems [5-7]. *Shewanella* species are able to respire a wide variety of organic and inorganic compounds, including oxygen, fumarate, nitrate, nitrite, thiosulfate, elemental sulfur, trimethylamine N-oxide, dimethyl sulfoxide (DMSO), and anthraquinone-2,6-disulphonate, as well as both soluble and solid metals, such as iron, manganese, uranium, chromium, cobalt, technetium, and vanadium [8-11]. This respiration electron acceptor plasticity implies that members of this genus have evolved flexible respiratory mechanisms in order to survive in redox-stratified environments, such as oxic/anoxic interfaces in sediments. Supporting this speculation, comparative genomic analysis among *Gammaproteobacteria* revealed that *Shewanella* have a relatively large number of signal-transduction proteins containing PAS domains, which are involved in the detection of various environmental signals, such as light, oxygen, and redox potential [12,13], suggesting that they have well-developed environment-sensing and regulatory systems. However, little is known about how *Shewanella* species regulate respiratory activity at the molecular level in response to changes in environmental conditions.

*S. oneidensis* MR-1 is the most extensively studied strain of *Shewanella* because of its annotated genome sequence [14], ease of genetic manipulation [5], and capability to directly transfer electrons to extracellular substances, such as metal oxides and electrodes, without exogenously added mediator [15]. Five primary component proteins, CymA, MtrA, MtrB, MtrC, and OmcA, comprising the extracellular electron transfer (EET) pathway (the Mtr respiratory pathway) have been identified in strain MR-1 [16]. OmcA and MtrC are outer membrane cytochromes (OM-cyts) containing 10 heme-binding sites, and play key roles in transferring electrons to extracellular electron acceptors [17]. It has been proposed that MR-1 releases electron from these OM-cyts through both direct EET pathways, in which electrons are directly transferred from OM-cyts that attach to solid metals [18,19], and indirect EET pathways, in which electrons are transferred from OM-cyts to distant solid metals via secreted electron-shuttle compounds, such as flavins [20,21]. Although biochemical studies indicate that both MtrC and OmcA are able to transfer electrons to solid Fe(III) oxides [18,19], MtrC appears to play a dominant role in electron transfer to electrodes, whereas OmcA plays an greater role in attachment of cells to solid surfaces [22,23], indicating that functional differences exist between these two OM-cyts.

**Figure 1:**
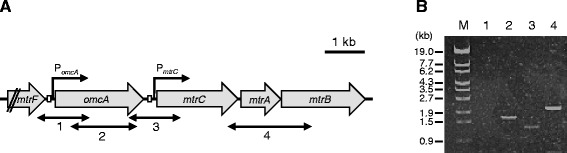
**The organization and transcriptional units of the** ***mtr*** **genes. (A)** Schematic illustration of the organization of the *mtr* genes. Solid arrows indicate the location and direction of the transcriptional promoters upstream of *omcA* (P_*omcA*_) and *mtrC* (P_*mtrC*_). The bidirectional arrows indicate the target regions of the RT-PCR analysis. The open boxes indicate the CRP-binding regions identified by the EMSA analysis. **(B)** RT-PCR analysis of the *mtr* genes. WT cells were grown anaerobically in LM containing 10 mM fumarate until the early stationary growth phase. The lane number corresponds to the target regions shown in panel **A**. The molecular sizes (kb) of the marker (lane M) are indicated to the left of the gel.

Despite extensive biochemical characterization of MtrC and OmcA, limited information is available on how MR-1 regulates these OM-cyt genes at the transcriptional level. In the MR-1 genome, four genes encoding the proteins comprising the Mtr respiratory pathway are organized in a cluster oriented in the same direction (Figure 1A). Previous studies of MR-1 have demonstrated that a cyclic AMP (cAMP) receptor protein (CRP) and adenylate cyclase (CyaC) responsible for cAMP production play key roles in transcriptional activation of the *mtr* genes, as well as the anaerobic respiratory genes involved in nitrate, fumarate, and DMSO reduction [24,25]. Although these genes are up-regulated under anaerobic (oxygen-limited) and electrode-respiring conditions [26-30], the molecular mechanisms and signal transduction pathways underlying the cAMP/CRP-dependent transcriptional activation of the *mtr* genes remain to be elucidated, as CRP does not contain PAS or other known redox-sensing domains. In addition, although two different transcription start sites (TSPs) have been identified in the upstream regions of *omcA* and *mtrC* [31,32], the regulatory mechanisms, including the role of CRP, in the transcription of the *mtr* genes have not been determined.

In the present study, we investigated the regulatory mechanisms that control expression of the *mtr* genes, particularly focusing on regulatory differences between *omcA* and *mtrC* and the involvement of CRP in the regulation of these genes. The findings presented here provide new insight into the complex regulatory mechanisms of the Mtr respiratory pathway in *Shewanella*.

## Results

### Transcriptional units of the *mtr* genes

**Figure 2:**
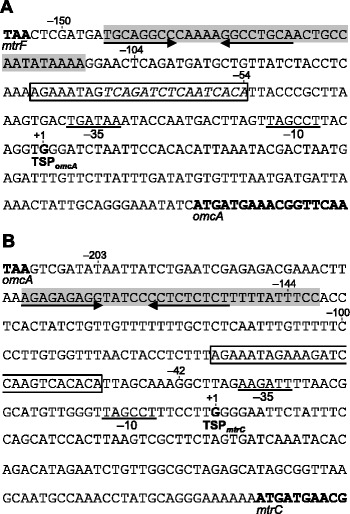
**Intergenic sequences upstream of** ***omcA*** **(A) and** ***mtrC*** **(B).** The positions of TSP_*omcA*_ and TSP_*mtrC*_ are shown. The nucleotides are numbered relative to each TSP (+1) and the positions of the 5′ ends of the pME series plasmids (Table 1) are indicated above the nucleotide sequences. Boxed sequences indicate the conserved sequences found upstream of the TSPs. A putative CRP-binding motif is italicized, putative −10 and −35 promoter sequences are underlined, and putative transcriptional terminator sequences predicted by the GeSTer program [33] are shaded. The arrows indicate palindromic sequences in the predicted terminators.

Previous studies have identified two independent TSPs for the *mtr* genes [31,32] (Figure 2). Beliaev et al. [31] detected a TSP located 119 bp upstream of the ATG start codon of *mtrC* (TSP_*mtrC*_; Figure 2B) using 5′ RACE PCR, and more recently, Shao et al. [32] identified a TSP located 93 bp upstream of *omcA* (TSP_*omcA*_; Figure 2A) among the total of 2,531 TSPs detected in *S. oneidensis* MR-1 using 5′-end RNA sequencing. Although these experiments were conducted using cells grown under aerobic [32] and unclear [31] conditions, the present 5′-RACE PCR analysis detected the identical TSPs (TSP_*omcA*_ and TSP_*mtrC*_) in both aerobically grown cells and cells cultured anaerobically with 10 mM fumarate. The presence of TSP_*mtrC*_ was also confirmed by primer extension analysis (Additional file 1: Figure S1). The 5′ RACE PCR using the *mtrA*- and *mtrB*-specific primers detected no TSPs other than TSP_*mtrC*_ (data not shown), indicating that *mtrC*, *mtrA*, and *mtrB* were co-transcribed as an operon, as suggested by Beliaev et al. [31].

A common sequence (5′-TAGCCT-3′) that was weakly similar to the *Escherichia coli* consensus −10 sequence (5′-TATAAT-3′) was present upstream of TSP_*omcA*_ and TSP_*mtrC*_, although a consensus −35 sequence was not conclusively identified upstream of either gene (candidate sequences are shown in Figure 2). Notably, highly conserved sequences were found in the upstream regions of TSP_*omcA*_ (5′-*AGAAATAG*TC*AGATC*TC*A*A*TCACA*-3′; position −77 to −54) and TSP_*mtrC*_ (5′-*AGAAATAG*AA*AGATC*CA*A*G*TCACA*-3′; position −76 to −53; common nucleotides are italicized) (Figure 2). The former sequence contained a putative CRP-binding motif (5′-TCAGATCTCAATCACA-3′) [34] based on its similarity to the consensus sequence for CRP binding in *E. coli* (5′-TGTGA-N6-TCTCA-3′) [35-37], suggesting that CRP directly binds to the DNA region upstream of TSP_*omcA*_. However, the corresponding sequence in the upstream region of TSP_*mtrC*_ (5′-AAAGATCCAAGTCACA-3′) contained only half of the CRP-binding consensus sequence.

We also performed RT-PCR analysis to investigate the transcriptional units of the *mtr* gene cluster using total RNA extracted from MR-1 cells grown anaerobically with 10 mM fumarate (Figure 1). No transcripts were detected when the intergenic region between *mtrF* and *omcA* was analyzed (Figure 1B, lane 1), suggesting that the transcription of *mtrF* is terminated by the putative terminator sequence downstream of *mtrF* (Figure 2A) and does not affect the transcription of *omcA*. Although a terminator-like sequence was also predicted downstream of *omcA* (Figure 2B), a weak band was detected when the *omcA*–*mtrC* intergenic region was amplified (Figure 1B, lane 3), indicating that the transcription from TSP_*omcA*_ is not completely terminated within this intergenic region. A transcript containing the *mtrC*–*mtrA* and *mtrA*–*mtrB* intergenic regions was also amplified (Figure 1B, lane 4), demonstrating the polycistronic transcription of these three genes.

### Differential expression of *omcA* and *mtrC*

**Figure 3:**
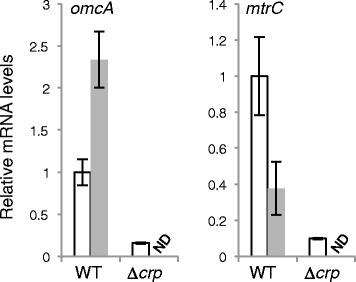
**qRT-PCR analyses of** ***omcA*** **and** ***mtrC*** **in WT and ∆** ***crp*** **cells.** Cells were grown in LM with oxygen (white bars) or 10 mM fumarate (light gray bars) as an electron acceptor until the early stationary growth phase. Results are expressed as relative values to mRNA levels in the WT cells grown with oxygen. The error bars represent the standard deviation calculated from at least three measurements. ND, not determined; expression levels under fumarate-reducing conditions were not determined in ∆*crp* because the cells did not grow under anaerobic conditions.

The identification of TSP_*omcA*_ and TSP_*mtrC*_ suggested that *omcA* and *mtrC* (the *mtrCAB* operon) were regulated by different regulatory mechanisms, leading to different expression patterns. To test this hypothesis, MR-1 cells were grown aerobically and under anaerobic conditions with 10 mM fumarate until the early stationary phase, and the expression levels of *omcA* and *mtrC* were then determined by quantitative RT-PCR analysis (Figure 3). The expression level of *omcA* under anaerobic conditions was 2.3-fold higher than that under aerobic conditions, whereas the expression level of *mtrC* under anaerobic conditions was 2.7-fold lower than that under aerobic conditions. We also measured the expression levels of *omcA* and *mtrC* in an in-frame *crp-*deletion mutant (∆*crp*). When ∆*crp* cells were grown under aerobic conditions (∆*crp* did not grow under fumarate-reducing conditions), the expression levels of *omcA* and *mtrC* were markedly decreased as compared with wild-type MR-1 (WT) cells (Figure 3). Taken together, these results demonstrate that, although CRP is essential for the transcriptional activation of both *omcA* and *mtrC*, these genes are differently regulated in response to culture conditions.

**Figure 4:**
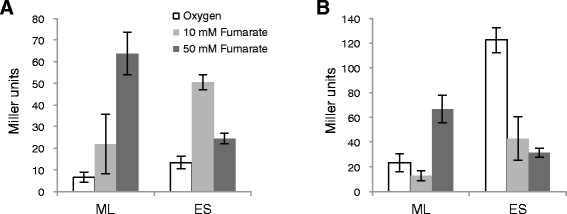
**LacZ expression from P**_***omcA***_ **(A) and P**_***mtrC***_ **(B).** WT cells harboring pMEomcA-150 or pMEmtrC-203 were grown with oxygen (white bars), 10 mM fumarate (light gray bars) or 50 mM fumarate (dark gray bars) as the electron acceptor, and their LacZ activities were measured in the mid-logarithmic (ML) and early stationary (ES) growth phases. The error bars represent the standard deviation calculated from at least three measurements. The LacZ activity in WT cells harboring pMElacZ (control vector) was below 3 Miller units under all of the tested conditions (data not shown).

To further characterize the expression patterns of *omcA* and *mtrC*, we constructed the *lacZ* reporter plasmids pMEomcA-150 and pMEmtrC-203 (Table 1), which contained nearly all of the intergenic regions upstream of *omcA* and *mtrC*, respectively (Figure 2), and compared the activity of promoters for *omcA* (P_*omcA*_) and *mtrC* (P_*mtrC*_) in WT cells under several different growth conditions (Figure 4). In this experiment, 10 and 50 mM fumarate were used for anaerobic cultivation to expose cells to electron acceptor- and electron donor-limited conditions, respectively (15 mM lactate was used as the electron donor), as it was reported that the electron donor to acceptor ratio affects the *c*-type cytochrome content and EET activities of MR-1 cells [38]. When cells were grown anaerobically with 10 and 50 mM fumarate, similar patterns of *lacZ* expression from P_*omcA*_ (Figure 4A) and P_*mtrC*_ were observed (Figure 4B). Specifically, under 10 mM fumarate-added conditions, the activities of both promoters were increased in the early stationary phase compared to those in the mid-logarithmic phase, whereas the opposite trend was observed under 50 mM fumarate-added conditions. In contrast, under aerobic growth conditions, P_*mtrC*_ activity was highest in the early stationary phase (Figure 4B), whereas P_*omcA*_ activity was only slightly elevated (Figure 4A). These results suggest that P_*omcA*_ and P_*mtrC*_ exhibit differential transcriptional responses in the presence of oxygen, but respond similarly to changes in the ratios of electron donors and acceptors under anaerobic conditions. The ratios of the expression levels under aerobic and 10 mM fumarate-added conditions were in good agreement with those observed in the qRT-PCR analysis (Figure 3), indicating that transcription from P_*omcA*_ does not significantly affect expression of the downstream *mtrCAB* operon *in vivo*. It is therefore likely that *omcA* transcription is largely attenuated due to the putative terminator sequence downstream of *omcA* (Figure 2B).

**Table 1:** **Bacterial strains and plasmids used in this study**

**Strain or plasmid**	**Relevant characteristic**	**Source or reference**
**Bacterial strains**		
*Escherichia coli*		
JM109	Host for cloning; *recA1. endAl, gyrA96, thi. hsdR17, supE44, relA1, λ-,* ∆(*lac-proAB*)*,* [F’*, traD36, proAB, lacI* ^q^ *Z*∆M15]	[39]
JM109λpir	Host for cloning pSMV10; JM109 lysogenized with λpir	[40]
WM6026	Donor strain for conjugation; *lacI* ^q^, *rrnB3*, DE*lacZ4787*, *hsdR514*, DE(*araBAD*)*567*, *E*(*rhaBAD*)*568*, *rph-1*, *att-lambda*::*pAE12-*del(*oriR6K-cat*::*frt5*), DE(*endA*)::*frt*, *uidA*(*delMluI*)::*pir*(wt), *attHK*::pJK1006-*del1/2* (del*oriR6K-cat*::*frt5,* del*trfA*::*frt*)	William Metcalf, University of Illinois
BL21 (DE3)	F^−^ *ompT hsdR17(r*_*B*_^*−*^ *m*_*B*_^*+*^ *) gal dcm*(DE3) F^−^, *ompT*, *hsdS*_B_(r_B_^−^ m_B_^−^), *gal*(λcI 857, *ind*1, *Sam*7, *nin*5, *lacUV*5-T7*gene*1), *dcm*(DE3)	Novagen
*Shewanella oneidensis*		
MR-1	Wild type	ATCC [2]
∆*crp*	The *crp* gene (SO_0624) disrupted	This study
**Plasmids**		
pMElacZ	pME4510 derivative, *lacZ* Gm^r^	[41]
pMEomcA-54	pMElacZ containing the region from −54 to +93 relative to TSP_*omcA*_	This study
pMEomcA-104	pMElacZ containing the region from −104 to +93 relative to TSP_*omcA*_	This study
pMEomcA-150	pMElacZ containing the region from −150 to +93 relative to TSP_*omcA*_	This study
pMEmtrC-42	pMElacZ containing the region from −42 to +119 relative to TSP_*mtrC*_	This study
pMEmtrC-100	pMElacZ containing the region from −100 to +119 relative to TSP_*mtrC*_	This study
pMEmtrC-144	pMElacZ containing the region from −144 to +119 relative to TSP_*mtrC*_	This study
pMEmtrC-203	pMElacZ containing the region from −203 to +119 relative to TSP_*mtrC*_	This study
pSMV10	9.1 kb mobilizable suicide vector; *oriR6K, mobRP4, sacB,* Km^r^, Gm^r^	Chad Saltikov, California Institute of Technology
pSMV-0624	1.5 kb fusion PCR fragment containing ∆*crp* cloned into the SpeI site of pSMV10	This study
pET-28(a)	Expression vector, T7 promoter	Novagen
pET-crp	pET-28(a) containing *N-ht-crp*	This study

### Upstream regulatory regions of P_*omcA*_ and P_*mtrC*_

**Figure 5:**
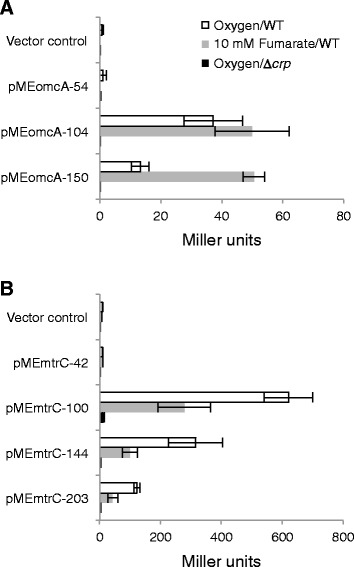
**5**′**-deletion analyses of the upstream regions of** ***omcA*** **(A) and** ***mtrC*** **(B).** WT or ∆*crp* cells harboring the indicated pME series plasmids were grown aerobically (WT, white bars; ∆*crp*, black bars) or anaerobically with 10 mM fumarate (WT, light gray bars), and their LacZ activities were measured in the early stationary growth phase. The error bars represent the standard deviation calculated from at least three measurements.

To determine the DNA regions involved in the transcriptional regulation of *omcA* and *mtrC*, we performed 5′-deletion analysis of the sequences upstream of TSP_*omcA*_ and TSP_*mtrC*_ in the reporter plasmids pMEomcA-150 and pMEmtrC-203 (Table 1). MR-1 strains harboring the reporter plasmids with promoter region deletions were grown aerobically or anaerobically with 10 mM fumarate until the early stationary phase, and LacZ activities were then measured and compared (Figure 5). Under aerobic growth conditions, the LacZ activity of cells transformed with pMEomcA-104, in which the DNA region from −150 to −105 relative to TSP_*omcA*_ was deleted, was increased to a comparable level to that in anaerobically grown cells (Figure 5A), suggesting the deleted DNA region was involved in the negative regulation of P_*omcA*_ under aerobic conditions. The LacZ activities of cells transformed with pMEomcA-54 under both aerobic and anaerobic conditions were decreased to the same level as those of pMElacZ (vector control), demonstrating that the region from −104 to −55 relative to TSP_*omcA*_, which contained the conserved upstream sequence and putative CRP-binding site (Figure 2A), was essential for the activation of P_*omcA*_.

In the 5′-deletion analysis of the sequence upstream of TSP_*mtrC*_ (Figure 5B), stepwise increases of LacZ activities were observed under both aerobic and anaerobic conditions when the DNA regions from −203 to −145 and from −144 and to −101 were deleted. However, the LacZ activities in the aerobically grown cells were consistently higher than those found in the anaerobically cultured cells. This finding suggests that the DNA region from −203 to −101 relative to TSP_*mtrC*_ contains sequences that constitutively repress the activity of P_*mtrC*_. As only low levels of LacZ activity was detected in cells harboring pMEmtrC-42, the region from −100 to −41, which also contained the conserved sequence upstream of TSP_*omcA*_ and TSP_*mtrC*_ (Figure 2B), was required for the activation of P_*mtrC*_.

### Direct activation of P_*omcA*_ and P_*mtrC*_ by CRP

**Figure 6:**
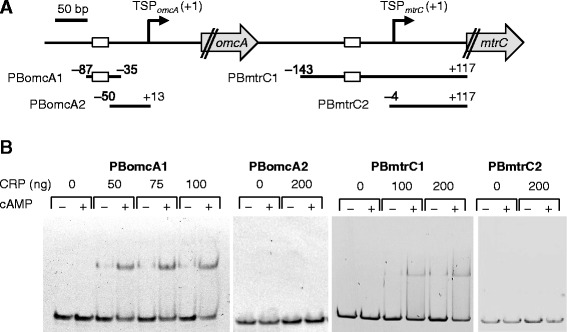
**Binding of CRP to the upstream regions of** ***omcA*** **and** ***mtrC*** **by EMSA. (A)** DNA fragments used as probes are indicated with bars. The positions of the 5′ and 3′ ends of the fragments relative to TSP_*omcA*_ or TSP_*mtrC*_ (+1) are shown. The open boxes indicate the consensus regions upstream of TSP_*omcA*_ or TSP_*mtrC*_ shown in Figure 2. **(B)** Binding of CRP to each probe in the presence (+) or absence (−) of cAMP. The probes were generated by PCR and labeled with Cy3 at the 5′ termini. The quantities of CRP are indicated above.

To investigate the involvement of CRP in the activation of P_*omcA*_ and P_*mtrC*_, we measured the LacZ activities of ∆*crp* cells transformed with the 5′-deletion reporter plasmids (Figure 5). No significant LacZ activity was detected for any of the reporter plasmids in ∆*crp* cells grown aerobically, demonstrating that CRP was essential for the activation of both P_*omcA*_ and P_*mtrC*_. We also performed electrophoretic mobility shift assays (EMSA) to investigate whether CRP directly binds to the regions upstream of *omcA* and *mtrC* (Figure 6). When a labeled DNA probe containing the region from −87 to −35 relative to TSP_*omcA*_ (PBomcA1; Figure 6A) was incubated with purified CRP protein, shifted bands corresponding to CRP-DNA complexes were observed in a cAMP-dependent manner (Figure 6B). However, no shifted bands were detected when a probe containing the region from −50 to +13 relative to TSP_*omcA*_ (PBomcA2) was used (Figure 6B), demonstrating that the sequence between −87 to −50, which contained the conserved upstream sequence and putative CRP-binding site (Figure 2), was required for the specific binding of CRP to the upstream region of TSP_*omcA*_. In EMSA performed with probes containing upstream regions of *mtrC* (PBmtrC1 [−143 to +117] and PBmtrC2 [−4 to +117]; Figure 6A), cAMP-dependent shifted bands were observed with PBmtrC1, but not with PBmtrC2, demonstrating that CRP can bind to the region from −143 to −4 relative to TSP_*mtrC*_ (Figure 6B). The weaker intensities of the shifted bands observed with PBmtrC1 compared with those with PBomcA1 are likely attributable to the lack of an obvious CRP-binding motif upstream of TSP_*mtrC*_ (Figure 2B). Taken together, these results indicate that CRP directly binds to the upstream regions of TSP_*omcA*_ and TSP_*mtrC*_, and up-regulates the transcription of the corresponding genes.

## Discussion

In this study, we demonstrated that two independent transcriptional promoters, P_*omcA*_ and P_*mtrC*_, differentially regulate the expression of *omcA* and *mtrC*, although both promoters require CRP for transcriptional activation. CRP is a global transcriptional regulator that plays a key role in regulating carbon metabolism in enteric bacteria [42,43]. In *Shewanella*, evidence suggests that CRP is involved in the regulation of anaerobic respiration [24,25,44]. For instance, the cAMP/CRP-dependent regulatory system was reported to be essential for regulating anaerobic arsenate reduction in *Shewanella* sp. strain ANA-3 [43]. CRP appears to be required for transcriptional activation of the genes involved in the reduction of Fe(III), Mn(IV), fumarate, nitrate, and DMSO in *S. oneidensis* MR-1, as a *crp*-deletion mutant of this strain exhibited decreased ability to reduce these electron acceptors [24]. Consistent with this finding, markedly reduced expression of several anaerobic respiratory genes (*mtr*, *fccA*, *nap*, and *dms*) were also observed in *crp* knockout mutants of MR-1 [25,45], although it remains unclear whether CRP directly or indirectly regulates these genes. In the present study, we confirmed that the expression from P_*omcA*_ and P_*mtrC*_ was markedly decreased in a *crp*-deletion mutant (∆*crp*) by qRT-PCR and LacZ reporter analyses (Figures 3 and 5). In addition, EMSA experiments revealed that CRP directly binds to the regions upstream of *omcA* and *mtrC* (Figure 6), demonstrating that the CRP/cAMP-dependent regulatory system directly activates both P_*omcA*_ and P_*mtrC*_. Taken together with the results of the 5′-deletion analysis (Figure 5), it is likely that the conserved sequences found upstream of TSP_*omcA*_ and TSP_*mtrC*_ (Figure 2) are critical for CRP binding and transcriptional initiation at these TSPs.

The mechanisms underlying the CRP/cAMP-dependent regulatory system have been extensively studied in *E. coli* K-12. When complexed with cAMP, CRP binds to a target DNA sequence, resulting principally in the transcriptional activation of the downstream gene [36,46]. The cAMP–CRP complex binds as a dimer to the consensus sequence 5′-TGTGA-N6-TCACAA-3′, which is typically found in the flanking regions (−35 and −10 sequences) of associated core promoters, and activates transcription by interacting directly with RNA polymerase [36,46-48]. Although CRP-dependent promoters exhibit great structural diversity, they are classified based on the position of the central base of the CRP-binding site [36]. The putative CRP-binding site upstream of TSP_*omcA*_ (5′-TCAGATCTCAATCACA-3′) and the corresponding site in the conserved sequence upstream of TSP_*mtrC*_ (5′-AAAGATCCAAGTCACA-3′) are centered at −61.5 and −60.5 relative to the respective TSPs, positions that closely correspond to those of the CRP-binding sites for class I CRP-dependent promoters (−61.5) [49,50]. Although P_*omcA*_ and P_*mtrC*_ lack a typical −35 sequence, it is known that −35 sequences are poorly conserved in most CPR-dependent promoters, whereas their −10 regions show relatively high similarity to the consensus sequence [36,51]. It is therefore conceivable that the cAMP–CRP complex in *S. oneidensis* activates transcription at P_*omcA*_ and P_*mtrC*_ in a similar manner to the well-characterized mechanism in *E. coli*, although the CRP target sequences appear to differ between these bacteria. A previous study reported that the addition of cAMP to aerobic cultures of MR-1 resulted in significant induction of fumarate reductase activity [24], suggesting that intracellular cAMP concentration is a key determinant of the ability of MR-1 cells to reduce anaerobic electron acceptors. However, although *E. coli* cells grown under fermentation or anaerobic respiration conditions produce more cAMP than cells grown under aerobic conditions [52], it is currently unknown how intracellular cAMP concentrations are regulated in *Shewanella*. Further investigation is therefore needed to elucidate the signal transduction mechanisms underlying the CRP/cAMP-dependent activation of P_*omcA*_ and P_*mtrC*_.

Similar transcriptional responses were observed for P_*omcA*_ and P_*mtrC*_ when cells were grown under fumarate-reducing conditions (Figure 4). However, the transcriptional activities of these promoters were dependent on the concentration of fumarate in the culture medium, suggesting that the balance of electron donors and acceptors strongly influences the activities of P_*omcA*_ and P_*mtrC*_. In medium supplemented with 10 mM fumarate, cells experience electron acceptor limitation during the stationary growth phase, because the fumarate is completely oxidized by the 15 mM lactate in the growth medium. The increased activities of P_*omcA*_ and P_*mtrC*_ under these conditions (Figure 4) suggest that the *mtr* genes are up-regulated in response to electron acceptor-limiting conditions. In contrast, when cells were grown with excess electron acceptor (50 mM fumarate), the activities of P_*omcA*_ and P_*mtrC*_ were decreased in the early stationary phase, suggesting that the *mtr* genes are down-regulated following the depletion of electron donors under anaerobic conditions. These notions are supported by the previous observation that MR-1 shows higher reduction activity toward an extracellular substance (azo dye) when grown in continuous culture under electron acceptor-limited conditions compared to electron donor-limited conditions [38]. It is therefore conceivable that the ratio of electron acceptors to electron donors influences intracellular redox status, such as the redox states of the NAD(H) and quinone pools [53], resulting in the altered expression of the *mtr* genes. However, in mid-logarithmic phase, the activities of P_*omcA*_ and P_*mtrC*_ in cultures containing 50 mM fumarate were higher than those in cultures supplemented with 10 mM (Figure 4). Although the reason for this difference is not clear, it is possible that other factors, such as cell density, determine the transcriptional activities of P_*omcA*_ and P_*mtrC*_ in the logarithmic growth phase, in which sufficient electron donors and acceptors are readily available.

The transcriptional activities of P_*omcA*_ and P_*mtrC*_ differed in the presence of oxygen (Figures 3 and 4). Although the *mtr* genes exhibit increased expression under anaerobic conditions compared with aerobic cultures [27,28], here, we found that cells grown under aerobic conditions until the early stationary phase displayed markedly higher transcriptional activity of P_*mtrC*_ compared to that observed under fumarate-reducing conditions (Figure 4B). In the mid-logarithmic phase, however, the activity of P_*mtrC*_ in the presence of oxygen was lower than that in anaerobic cultures supplemented with 50 mM fumarate, but was slightly higher than that under the 10 mM fumarate-added condition, indicating that the ratios of aerobic and anaerobic expression from P_*mtrC*_ are strongly influenced by the growth phase and anaerobic electron acceptor concentration. In contrast, the activity of P_*omcA*_ was maintained at lower levels than that of P_*mtrC*_ under aerobic conditions (Figure 4A), demonstrating a clear difference in the regulation of P_*omcA*_ and P_*mtrC*_. This finding suggests that although P_*omcA*_ and P_*mtrC*_ are both dependent on CRP, additional mechanisms are also likely involved in the regulation of these promoters. Notably, the activity of P_*omcA*_ in aerobically grown cells was increased by deleting the DNA region from −150 to –104 relative to TSP_*omcA*_, suggesting that this region is involved in the negative regulation of P_*omcA*_. In *E. coli*, the CytR regulator acts as a transcriptional repressor for several CRP-dependent promoters, including *deoP2*, by binding to an operator region located upstream of a CRP-binding site (CRP-1) through protein-protein interactions with the cAMP-CRP complex, resulting in transcriptional repression [54,55]. Therefore, it is possible that under aerobic conditions, an unknown regulator binds to the upstream region of P_*omcA*_ and represses the transcription of *omcA*.

5′-deletion analysis demonstrated that deletion of the DNA regions upstream of P_*mtrC*_ significantly increased the activity of this promoter under both aerobic and anaerobic conditions. Although the reason for this increase remains unclear, it is possible that a non-specific repressor, such as a homolog of *E. coli* H-NS [56], which binds preferentially to AT-rich DNA sequences, binds to the upstream region of P_*mtrC*_ and constitutively represses expression from this promoter. This speculation is supported by the fact that positions −203 to −101 relative to TSP_*mtrC*_ have a very low GC content, and that relationships between H-NS and CRP-dependent promoters have been found in *E. coli* [57,58].

## Conclusions

The results of the present study indicate that transcription of the *mtr* genes is regulated by the CRP/cAMP-dependent regulatory system and yet-unidentified negative regulatory mechanisms, suggesting that *Shewanella* exploit multiple signal transduction pathways to control EET activity in response to environmental conditions and intracellular energy status. The differential expression of *omcA* and *mtrC* under aerobic conditions (Figure 4) indicates that redox-sensing regulators, such as PAS domain-containing proteins, are involved in the expression of these OM-cyt genes, thereby tuning the composition of the OM-cyts to the environment. As the expression of *mtrC* under aerobic conditions was markedly up-regulated in the early stationary phase, growth phase-dependent regulatory mechanisms might also be involved in *mtr* gene transcription. Further studies are needed to elucidate the molecular mechanisms underlying the regulatory differences between P_*omcA*_ and P_*mtrC*_, and to explore the functional relationship between the OM-cyts of *Shewanella*.

## Methods

### Bacterial strains, plasmids, and growth conditions

The bacterial strains and plasmids used in this study are listed in Table 1. *E. coli* strains were routinely cultured in Luria-Bertani (LB) medium or 2× yeast extract-tryptone (2× YT) medium at 37°C. *E. coli* mating strain (WM6026) required 2,6-diaminopimelic acid (DAP) at 100 μg/mL for growth. *S. oneidensis* strains were cultured at 30°C in LB medium or modified lactate medium (LM) comprised of 15 mM lactate, 9 mM (NH_4_)_2_SO_4_, 5.7 mM K_2_HPO_4_, 3.3 mM KH_2_PO_4_, 5.0 g/L yeast extract, and 30 mM HEPES-NaOH buffer (pH 7.4). LM was supplemented with yeast extract to supply cells with abundant nutrients, other than electron donors and acceptors. The optical density at 600 nm (OD_600_) of the cultures was measured using a DU800 spectrophotometer (Beckman). For aerobic cultivation, *S. oneidensis* strains were inoculated in 300-mL baffled Erlenmeyer flasks containing 100 mL LM, and were cultivated with shaking on a rotary shaker at 180 rpm until the middle logarithmic or early stationary growth phase (OD_600_ 0.5 to 0.7, or >2.0, respectively). Fumarate was used as a model anaerobic electron acceptor in this study because it is known that *S. oneidensis* MR-1 requires CRP to reduce both solid metals and fumarate [24], and exhibits similar transcriptional responses when exposed to metal and non-metal anaerobic electron acceptors [27]. For anaerobic cultivation under electron acceptor-limited conditions, *S. oneidensis* strains were inoculated in 100-mL bottles containing 80 mL LM supplemented with 10 mM fumarate, and were cultured until the middle logarithmic or early stationary growth phase (OD_600_ of 0.08 to 0.1, or >0.25, respectively). For anaerobic cultivation under electron donor-limited conditions, *S. oneidensis* strains were cultured in LM supplemented with 50 mM fumarate until the middle logarithmic or early stationary growth phase (OD_600_ of 0.2 to 0.4 or >0.7, respectively). The bottles containing the anaerobic cultures were capped with Teflon-coated butyl rubber septum, sealed with aluminum crimp seals, and purged with pure nitrogen gas. When necessary, 100 μg/mL ampicillin, 15 μg/mL gentamicin (Gm), or 50 μg/mL kanamycin (Km) was added to the culture medium. Agar plates contained 1.5% Bacto agar (Difco).

### RNA extraction

*Shewanella* cells were grown aerobically in LM or anaerobically in LM containing 10 mM fumarate, and were harvested at early stationary growth phase. RNA was extracted from cells using Trizol reagent (Invitrogen) following the manufacturer’s instructions and was purified using an RNeasy Mini Kit and RNase-Free DNase Set (Qiagen). The quality of extracted RNA was evaluated using an Agilent 2100 Bioanalyzer with RNA 6000 Pico reagents and RNA Pico Chips (Agilent Technologies) according to the manufacturer’s instructions.

### RT-PCR

For cDNA synthesis, 1.0 μg total RNA extracted from anaerobically grown MR-1 cells was subjected to a reverse transcription (RT) reaction using Superscript III Reverse Transcriptase (Invitrogen) and Random Primers (Invitrogen) following the manufacturer’s instructions. The cDNA was amplified with Ex Taq DNA polymerase (TaKaRa) and the primer sets listed in Additional file 2: Table S1. Amplification conditions were as follows: an initial denaturation of 95°C for 30 s, followed by 25–30 amplification cycles consisting of 95°C for 30 s, 55°C for 30 s and 72°C for 30 s, and a final elongation at 72°C for 7 min. Negative control reactions without reverse transcriptase were also performed.

### Determination of transcription start points

5′-Rapid amplification of cDNA ends (5′-RACE) PCR reactions were performed using 1.0 μg total RNA extracted from anaerobically grown MR-1 cells and a SMATer RACE cDNA Amplification Kit (Clontech) following the manufacturer’s instructions. The first-strand cDNA was synthesized using the gene-specific primer omcA_RACE_out or mtrC_RACE_out (Additional file 2: Table S1) and subsequently amplified using Universal Primer A Mix (Clontech) and the nested primer omcA_RACE_in or mtrC_RACE_in (Additional file 2: Table S1). The amplified fragments were analyzed by agarose gel electrophoresis and purified using a QIAquick PCR purification kit (Qiagen). The purified fragments were cloned into T-Vector pMD19 (Takara) and sequenced to determine the 5′-end points.

Primer extension analysis was performed according to a previously described method with slight modifications [59]. Briefly, 10 μg total RNA extracted from aerobically or anaerobically grown MR-1 cells was reverse-transcribed using SuperScript III Reverse Transcriptase (Invitrogen) and an IR800-labeled primer (Aloka), PE-mtrC-100 (Additional file 2: Table S1). The primer extension products were purified by phenol-chloroform extraction and ethanol precipitation, and were then subjected to electrophoresis using a Li-Cor 4200 Automated DNA Sequencer (Li-Cor) together with the sequence reaction products generated with the same primer.

### qRT-PCR

Quantitative RT-PCR (qRT-PCR) was performed using a LightCycler 1.5 instrument (Roche) according to a method described previously [60-62]. The PCR mixture (20 μL) contained 15 ng total RNA, 1.3 μL of 50 mM Mn(OAc)_2_ solution, 7.5 μL LightCycler RNA Master SYBR Green I (Roche), and 0.15 μM of the primers listed in Additional file 2: Table S1. To generate standard curves, DNA fragments of target genes (*mtrC, omcA*, and the 16S rRNA gene) were PCR amplified from the total DNA of strain MR-1, and were then purified by agarose gel electrophoresis using a QIAEX II Gel Extraction Kit (Qiagen) following the manufacturer’s instructions. A dilution series of the purified products from each reaction and the original RNA samples were used as template for quantitative PCR analysis. Specificity of the quantitative PCR was verified by dissociation-curve analysis. The expression levels of the target genes (*mtrC* and *omcA*) were normalized based on the expression level of the reference gene (16S rRNA gene). All measurements were performed in triplicate at a minimum.

### Gene disruption

The in-frame disruption of the *crp* gene in strain MR-1 was performed using a two-step homologous recombination method with suicide plasmid pSMV-10, as described previously [63-65]. Briefly, a 1.6-kb fusion product, consisting of upstream (746 bp) and downstream (724 bp) sequences of the *crp* gene joined by an 18-bp linker sequence, was constructed by PCR and *in-vitro* extension using the primers listed in Additional file 2: Table S1. The amplified fusion product was ligated into the SpeI site of pSMV-10, generating pSMV-crp, which was then introduced into MR-1 by filter mating with *E. coli* WM6026. Transconjugants (single-crossover clones) were selected on LB plates containing Km and were further cultivated for 20 h in LB medium lacking antibiotics. The cultures were then spread onto LB plates containing 10% (w/v) sucrose to isolate Km-sensitive double-crossover mutants. Disruption of the *crp* gene in the obtained strains was confirmed by PCR. One representative mutant strain in which the *crp* gene was disrupted in-frame was selected and designated *∆crp*.

### β-Galactosidase reporter assay

To construct a series of P_*omcA*_*-lacZ* transcriptional fusion plasmids (Table 1), the regions upstream of *omcA* were amplified from total DNA of MR-1 using a series of forward primers (omcA_F-54 through omcA_F-150) and a reverse primer, omcA_R + 93. To construct a series of P_*mtrC*_-*lacZ* transcriptional fusion plasmids (Table 1), a series of forward primes (mtrC_F-42 through mtrC_F-203) and a reverse primer, mtrC_R + 117, were used. PCR products were digested with EcoRI and HindIII, or BamHI and HindIII (restriction endonuclease sites incorporated into the primer sequences; see Additional file 2: Table S1), and the fragments were cloned between the corresponding sites of pMElacZ [41]. Positive clones were verified by DNA sequencing. The constructed reporter plasmids were introduced into wild-type MR-1 or ∆*crp* by electroporation according to a method described elsewhere [66]. The resultant reporter strains were aerobically or anaerobically grown in LM as described above. β-Galactosidase activity was measured in triplicate at a minimum according to the method of Miller [67].

### CRP protein purification

To construct a plasmid expressing N-terminally histidine-tagged CRP (N-ht-CRP) protein, the *crp* gene was amplified from total DNA of MR-1 with primers crp_NdeI_F and crp_BamHI_R (Additional file 2: Table S1). The obtained PCR products were digested with NdeI and BamHI, and cloned between the corresponding sites of the expression vector pET-28a(+) (Novagen), which contains an N-terminal histidine tag sequence. The resultant plasmid, pET-N-ht-crp (Table 1), was introduced into *E. coli* BL21(DE3). Cells carrying the plasmid were grown in 300-mL baffled Erlenmeyer flasks containing 100 mL 2× YT medium supplemented with Km at 30°C. Isopropyl-1-thio-β-D-galactopyranoside (IPTG) was added to a final concentration of 0.1 mM when the OD_600_ reached 0.5 to 0.8. After further cultivation for 3 h, the cells were harvested by centrifugation, washed with IMAC wash buffer (Bio-Nobile), and suspended in 3.0 mL of the same buffer. The cell suspension was subjected to ultrasonication with a Misonix S4000 Sonicator (Misonix). The cell extract was centrifuged at 1000 × *g* for 5 min to remove cell debris. N-ht-CRP contained in the supernatant was purified using a QuickPick IMAC Metal Affinity Kit for Proteins (Bio-Nobile) following the manufacturer’s instructions. The protein samples eluted by the QuickPick IMAC system were analyzed by sodium dodecyl sulfate-polyacrylamide gel electrophoresis (SDS-PAGE) and stored at 4°C until use (within 12 h). The protein concentration was determined using a Micro BCA Protein Assay Kit (Pierce).

### EMSA

Cy3-labeled DNA probes were generated by PCR with the Cy3-labeled primer sets listed in Additional file 2: Table S1. PCR products were analyzed by non-denaturing polyacrylamide gel electrophoresis and then purified using a QIAEX II Gel extraction Kit (Qiagen) following the manufacturer’s instructions. An electrophoretic mobility shift assay (EMSA) was performed as previously described, but with slight modifications [41]. DNA-binding reactions were performed in a 20-μL reaction mixture containing 10 mM Tris–HCl (pH 7.6), 0.5 mM disodium EDTA (pH 8.0), 100 mM KCl, 50 μg/mL bovine serum albumin, 50 μg/mL poly (deoxyinosinic-deoxycytidilic) acid [poly(dI-dC); Sigma-Aldrich], 50 μM cAMP, 10% (v/v) glycerol, 2 nM Cy3-labeled DNA probe, and 0 to 200 ng of purified N-ht-CRP. The mixture was incubated on ice for 30 min and then loaded onto a non-denaturing 12.5% polyacrylamide gel. Electrophoresis was conducted at 150 V in 0.5× Tris-borate-EDTA (TBE) buffer. Fluorescent gel images were obtained using a Typhoon FLA 9000 (GE Healthcare).

## Additional files

Additional file 1: Figure S1. Determination of the transcription start point (TSP) upstream of *mtrC* by primer extension. The arrow indicates the primer extension product generated using equal amounts of total RNA from wild-type (WT) cells grown aerobically (lane 1) or anaerobically with 10 mM fumarate (lane 2). Lanes T, A, G, and C correspond to the sequence ladders generated with the same primer as the primer extension products; the sequence pattern is shown to the right of the gel. Additional file 2: Table S1. Primers used in this study.

## Abbreviations

CRP: cAMP receptor protein
DMSO: Dimethyl sulfoxide
EET: Extracellular electron transfer
OM-cyt: Outer-membrane cytochrome
TSP: Transcription start site
RACE: Rapid amplification of cDNA ends
WT: Wild-type
EMSA: Electrophoretic mobility shift assay
DAP: 2,6-diaminopimelic acid
LM: Lactate medium
N-ht-CRP: N-terminally histidine-tagged CRP
