# Supplementary figures and images for: Transcriptional mechanisms for differential expression of outer membrane cytochrome genes omcA and mtrC in Shewanella oneidensis MR-1
Source: BMC Microbiol. 2015 Mar 21;15:68. doi: 10.1186/s12866-015-0406-8 (PMC4417206; doi:10.1186/s12866-015-0406-8)

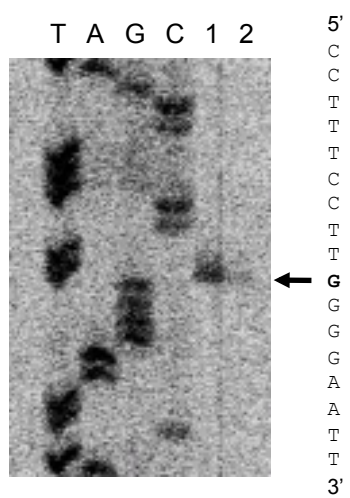

Supplement: Additional file 1: Figure S1. — Determination of the transcription start point (TSP) upstream of mtrC by primer extension. The arrow indicates the primer extension product generated using equal amounts of total RNA from wild-type (WT) cells grown aerobically (lane 1) or anaerobically with 10 mM fumarate (lane 2). Lanes T, A, G, and C correspond to the sequence ladders generated with the same primer as the primer extension products; the sequence pattern is shown to the right of the gel. [file 12866_2015_406_MOESM1_ESM.pdf]
