# Supplementary material for: Transcriptional mechanisms for differential expression of outer membrane cytochrome genes omcA and mtrC in Shewanella oneidensis MR-1
Source: BMC Microbiol. 2015 Mar 21;15:68. doi: 10.1186/s12866-015-0406-8 (PMC4417206; doi:10.1186/s12866-015-0406-8)
Supplement: Additional file 2: Table S1. — Primers used in this study. [file 12866_2015_406_MOESM2_ESM.pdf]

Table S1. Primers used in this study

Primer	Sequence (5' to 3')	Modification, for use
qRT-omcA-F	GGATACGGCGTTGAAGATGT	qRT-PCR for *omcA*
qRT-omcA-R	TGGTATCCGTTCCATTCCAT	qRT-PCR for *omcA*
qRT-mtrC-F	CGGCAATGATGGTAGTGATG	qRT-PCR for *mtrC*
qRT-mtrC-R	GCTTCGTTAGTGGCGAAAAC	qRT-PCR for *mtrC*
qRT-16S-F	AGCGCAACCCCTATCCTTAT	qRT-PCR for 16S rRNA gene
qRT-16S-R	CGTAAGGGCCATGATGACTT	qRT-PCR for 16S rRNA gene
PE-mtrC-100	CCATCACTACCATCATTGCC	5'-IR800, primer extention
RT-omcA-mtrF-F	TAACAATGGCACCTTCACCA	RT-PCR, region 1 (Fig. 1)
RT-omcA-mtrF-R	TCGCCACCTTTATGGATAGC	RT-PCR, region 1 (Fig. 1)
RT-omcA-F	CCATCAGGCGTTGATAACCT	RT-PCR, region 2 (Fig. 1)
RT-omcA-R	ATACCCAAATTACGGCACCA	RT-PCR, region 2 (Fig. 1)
RT-omcA-mtrC-F	GAAAGCCCACGAAAGTGAAG	RT-PCR, region 3 (Fig. 1)
RT-omcA-mtrC-R	CCTTCTACGTGGCAAGAAGC	RT-PCR, region 3 (Fig. 1)
RT-mtrCAB-F	AAGAAGAAAGGCGCATTGAA	RT-PCR, region 4 (Fig. 1)
RT-mtrCAB-R	GAGTGCGGTAAACCAACGAT	RT-PCR, region 4 (Fig. 1)
omcA_Race_in	CGGGAGTTAATTGCGCAATACCAAATCGC	5' RACE PCR
omcA_Race_out	CCGCGGTCAGCTTCTGTTTCTCCCAC	5' RACE PCR
mtrC_Race_in	GCGAGACCAATCACTGGCATGTCGGC	5' RACE PCR
mtrC_Race_out	CGCCCCTTCCGGTATTAATTGCAGTGC	5' RACE PCR
omcA_R+93	GTAG**AAGCTT**GATATTTCCCTGCAATAGTTTTAATCA	**HindIII**, LacZ reporter assay
omcA_F-54	GATG**GAATTC**TTACCCGCTTAAAGTGACTG	**EcoRI**, LacZ reporter assay
omcA_F-104	GTAC**GAATTC**CAGATGATGCTGTTATCTACCTC	**EcoRI**, LacZ reporter assay
omcA_F-150	GATC**GAATTC**ATGATGCAGGCCCAAAAG	**EcoRI**, LacZ reporter assay
mtrC_R+117	GATG**AAGCTT**TTTTCCCTGCATAGGTTTGG	**HindIII**, LacZ reporter assay
mtrC_F-42	CTCG**GGATCC**GCTTAGAAGATTTTAACGGCATGT	**BamHI**, LacZ reporter assay
mtrC_F-100	TGAC**GGATCC**CCTTGTGGTTTAACTACCTCTTTAGAA	**BamHI**, LacZ reporter assay
mtrC_F-144	CTAC**GGATCC**TCCACCTCACTATCTGTTGTTTTTTTGCTCTC	**BamHI**, LacZ reporter assay
mtrC_F-203	CTAC**GGATCC**AATTATCTGAATCGAGAGACGAAA	**BamHI**, LacZ reporter assay
*crp*_5'-out-SpeI	GG**ACTAGT**GGTCACGTTTAATCAACTGGCC	**SpeI**, *crp* disruption
*crp*_5'-in	**GCTGTTACCCTGAGCTGA**CAGAGCCATGTCGATGTTCCTCG	**Linker**, *crp* disruption
*crp*_3'-in	**TCAGCTCAGGGTAACAGC**CGTTAAGTTAGACTTCAGCTTG	**Linker**, *crp* disruption
*crp*_3'-out-SpeI	GG**ACTAGT**GCGCTAGCAATTCCTGCGCG	**SpeI**, *crp* disruption
crp_NdeI_F	CATG**CATATG**ATGGCTCTGATTGGTAAGCC	**NdeI**, pET-N-ht-crp
crp_BamHI_R	GATC**GGATCC**TTAACGGGTACCATATACCAC	**BamHI**, pET-N-ht-crp
omcA-50_F	CCCGCTTAAAGTGACTGATAAATACC	5'-Cy3, PBomcA2
omcA-87_F	CTACCTCAAAAGAAATAGTCAG	5'-Cy3, PBomcA1
omcA+13_R	GGAATTAGATCCCACCTGTAAGGC	5'-Cy3, PBomcA2
omcA-35_R	CAGTCACTTTAAGCGGGTAATG	5'-Cy3, PBomcA1
mtrC-4_F	CCTTGGGGAATTCTATTTCC	5'-Cy3, PBmtrC2
mtrC-143_F	TCCACCTCACTATCTGTTGTTTTTTTGCTCTC	5'-Cy3, PBmtrC1
mtrC+117_R	TTTTCCCTGCATAGGTTTGG	5'-Cy3, PBmtrC1, PBmtrc2
